# Different Respiratory Rates during Resuscitation in a Pediatric Animal Model of Asphyxial Cardiac Arrest

**DOI:** 10.1371/journal.pone.0162185

**Published:** 2016-09-12

**Authors:** Jorge López, Sarah N. Fernández, Rafael González, María J. Solana, Javier Urbano, Jesús López-Herce

**Affiliations:** 1 Pediatric Intensive Care Unit, Gregorio Marañón General University Hospital, Madrid, Spain; 2 School of Medicine Complutense University of Madrid, Madrid, Spain; 3 Gregorio Marañón Health Research Institute, Madrid, Spain; 4 Mother-Child Health and Development Network (RedSAMID) of Carlos III Health Institute, Bilbao, Spain; Centre Hospitalier Universitaire Vaudois, FRANCE

## Abstract

**Aims:**

Actual resuscitation guidelines recommend 10 respirations per minute (rpm) for advanced pediatric life support. This respiratory rate (RR) is much lower than what is physiological for children. The aim of this study is to compare changes in ventilation, oxygenation, haemodynamics and return of spontaneous circulation (ROSC) rates with three RR.

**Methods:**

An experimental model of asphyxial cardiac arrest (CA) in 46 piglets (around 9.5 kg) was performed. Resuscitation with three different RR (10, 20 and 30 rpm) was carried out. Haemodynamics and gasometrical data were obtained at 3, 9, 18 and 24 minutes after beginning of resuscitation. Measurements were compared between the three groups.

**Results:**

No statistical differences were found in ROSC rate between the three RR (37.5%, 46.6% and 60% in the 10, 20 and 30 rpm group respectively P = 0.51). 20 and 30 rpm groups had lower PaCO2 values than 10 rpm group at 3 minutes (58 and 55 mmHg vs 75 mmHg P = 0.08). 30 rpm group had higher PaO2 (61 mmHg) at 3 minutes than 20 and 10 rpm groups (53 and 45 mmHg P = 0.05). No significant differences were found in haemodynamics or tissue perfusion between hyperventilated (PaCO2 <30 mmHg), normoventilated (30–50 mmHg) and hypoventilated (>50 mmHg) animals. PaO_2_ was significantly higher in hyperventilated (PaO_2_ 153 mmHg) than in normoventilated (79 mmHg) and hypoventilated (47 mmHg) piglets (P<0.001).

**Conclusions:**

Our study confirms the hypothesis that higher RR achieves better oxygenation and ventilation without affecting haemodynamics. A higher RR is associated but not significantly with better ROSC rates.

## Introduction

Actual resuscitation guidelines are based on international consensus [1,2]. In the last few years, chest compressions have gained greater relevance than breaths during CPR [3], even to the point of recommending chest compressions only for bystander adult basic life support [4]. Nevertheless, experimental studies in animal models and clinical studies in children show that CPR with breaths and chest compressions achieves better oxygenation, ventilation, survival and neurological outcomes than CPR with chest compressions only [5–7].

This is probably due to the different aetiology of cardiopulmonary arrest and because illness and pathophysiological responses of paediatric patients often differ from those seen in adults. Cardiac arrest in children and young adults is usually the end result of an initial respiratory arrest (secondary to respiratory or neurological conditions), whereas the main cause of cardiac arrest in adults is cardiogenic due to arrhythmias [2,8–14]. This is why ventilation during CPR is more important in children than in adults [5–7,15].

International CPR guidelines for advanced life support recommend a chest compression rate of 100 to 120 compressions per minute (cpm) and 10 respirations per minute (rpm) for both adults and children [1,2,16]. Normal respiratory rate in children ranges from 40 rpm in neonates to 20 rpm in the older child [17], so the recommended 10 rpm during CPR is much lower than what is physiological for them. The theoretical reason for using low respiratory rates (RR) during CPR is that the pulmonary blood flow obtained from chest compressions is low, and thus low minute volumes should be sufficient for blood oxygenation and carbon dioxide (CO_2_) clearance [4].

These recommendations are also supported on the belief that hyperventilation during CPR with low arterial CO_2_ pressure (PaCO_2_) can cause cerebralvasoconstriction and compromise cerebral perfusion. Furthermore, hyperventilation can compromise systemic venous return and myocardial perfusion which, in addition to potential interruptions in chest compressions while delivering breaths, can contribute to a significant reduction in blood flow delivery to the tissues. Several studies in adult patients and in animal models find an association between hyperventilation and decreased coronary perfusion, survival and worse neurological outcomes [18–23].

On the other hand, other studies in adults and children show that both hyperventilation and hypoventilation after the return of spontaneous circulation (ROSC) are associated with higher mortality rates [24–26].

Actual recommendations emphasize the importance of reversing the main cause that led to cardiac arrest (CA) as soon as possible [2,27]. For instance, in the case of asphyxia, the causes that lead to CA are severe hypoxia and hypercapnia. In these cases, higher respiratory rates during CPR would achieve faster normalization of oxygenation and ventilation and, theoretically, facilitate ROSC.

There are not, to our knowledge, any clinical studies analyzing what the ideal RR during CPR is for children. The obvious difficulties in carrying out randomized clinical trials during CPR make it difficult to offer high quality scientific evidence [2]. For this reason, paediatric recommendations are based on studies with low quality scientific evidence, studies in adults and studies in non-asphyxial CA paediatric animal models [28]. This is why paediatric experimental animal models to assess the effect of ventilation during CPR are so important [6,7].

The hypothesis of the present study is that a RR of 10 rpm during CPR can be insufficient for smaller children, since the younger the child the higher RR is needed for adequate ventilation [17,29]. Thus, higher RR during CPR would achieve better oxygenation and ventilation without negatively affecting haemodynamics and improve ROSC rates.

## Materials and Methods

We conducted a randomized controlled experimental clinical trial in 46 Maryland piglets that were genetically identical. The study was approved by the Gregorio Marañón General University Hospital Ethics Committee for Animal Research (4-2/2012) and was carried out by qualified staff. International guidelines for ethical conduct in the care and use of experimental animals were applied throughout the study.

Animals were brought from a Community of Madrid authorised farm and housed for 24 hours before the experiment and were fasted overnight (with free access to water). Piglets were pre-medicated with intramuscular ketamine (15 mg/kg) and atropine (0.02 mg/kg) before obtaining a peripheral venous access. After starting continuous cardio-respiratory monitoring, a single dose of iv propofol (5 mg/kg), fentanyl (5 mcg/kg) and atracurium (0.5 mg/kg) were administered for orotracheal intubation, followed by a continuous intravenous perfusion of propofol (10 mg/kg/h), fentanyl (10 mcg/kg/h) and atracurium (2 mg/kg/h).

Piglets were mechanically ventilated (Servo 900C^®^ Ventilator, Siemens-Elema, Solna, Sweden) with the initial following settings: tidal volume 10 ml/kg, 20 bpm, PEEP 4 mmHg, FiO2 45%. Settings were adjusted to obtain an end-tidal CO_2_ (etCO_2_) between 30–40 mmHg and an arterial CO_2_ pressure between 35 and 45 mmHg.

Continuous monitoring of the following parameters were registered: electrocardiogram (ECG), transcutaneous oxygen saturation (HeartStart XL+^®^, Philips Medical Systems, Andover, Massachusetts, USA), cerebral blood flow by means of a flowmeter placed on the carotid artery (HDQ1.5FSB, Transonic Systems Inc., Ithaca, New York, USA), skin blood perfusion on the abdomen (BLF21A Laser Doppler Perfusion Monitor^®^, Transonic Systems Inc., Ithaca, New York, USA), regional oxygen saturation (rSO_2_) of cerebral and splanchnic regions (sensors in cranial midline and right flank, respectively) using near infrared spectroscopy (NIRS) (INVOS^®^ Cerebral Oxymeter Monitor, Somanetics, Troy, Minnesota, USA). Ventilating volumes and pressures, FiO_2_ and etCO_2_ were registered by means of a spirometer connected to an S5^®^ monitor (DatexOhmeda, Madison, Wisconsin, USA). Cannulation of femoral arterial and venous accesses was ultrasound-guided. A three-lumen 5F catheter was used for continuous central venous pressure (CVP) monitoring, blood sample extraction and drug infusion. A 4F PiCCO^®^catheter (PiCCO^®^, Pulsion Medical System, Munich, Germany) for monitoring arterial pressure and cardiac output byfemoral artery thermodilution method was placed in the contralateral femoral artery. Blood gas analyses were processed in a GEM Premier 300^®^ gas analyzer (Instrumentation Laboratory, Lexington, Kentucky, USA).

After a 30-minute stabilization period, baseline data were collected and arterial and venous blood gases were drawn to assess adequate ventilation and oxygenation.

Asphyxial cardiac arrest was induced by disconnecting the piglets from the ventilator for 10 minutes after receiving an additional bolus of atracurium (0.5 mg/kg), and cardiac arrest was defined as a mean arterial pressure (MAP) under 25mmHg, as has been described previously [6,7,30,31]. Data including monitoring parameters and blood gases were registered after 10 minutes of asphyxia, before starting resuscitation. Time to cardiac arrest was also registered. At this point, animals were randomized into one of the three therapeutic groups (10, 20 or 30 rpm) and advanced resuscitation was initiated: the animal was connected to the ventilator (with the same parameters as before the disconnection, except for an FiO_2_ of 100% and the allotted breath rate) and chest compressions were delivered at a metronome-tailored rate of 100 cpm. Pulse and ECG were assessed at 3 minute intervals, and the provider delivering chest compressions was swapped to avoid fatigue. Adrenaline (Epinephrine)(0.02 mg/kg each dose) was administered every 3 minutes and sodium bicarbonate (1 mEq/kg each dose) at 9 and 18 minutes of CPR. Animals were defibrillated (4 J/kg) if a shockable rhythm was identified; adrenaline and amiodarone (5 mg/kg) were administered after the third defibrillation [28].

The following data were collected at baseline and every 3 minutes after the initiation of CPR: Heart rate and rhythm, systolic arterial pressure (SAP), diastolic arterial pressure (DAP), mean arterial pressure (MAP), transcutaneous oxygen saturation, cerebral and splanchnic tissue oxygenation indexes, cerebral blood flow, skin blood perfusion, temperature, inspiratory and expiratory tidal volume, etCO_2_ and FiO_2_. Arterial and venous blood gases were drawn at baseline 3, 9, 18 and 24 minutes.

Resuscitation was discontinued upon ROSC or after 24 minutes of CPR. Animals achieving ROSC were later sacrificed by means of propofol and potassium chloride overdose.

Statistical package SPSS 21.0 (IBM SPSS Statistics, Chicago, Michigan, USA) was used for statistical analysis. Variables did not follow a normal distribution according to the Kolmogorov-Smirnov test. Continuous variables are expressed as medians and interquartile range (IQR), and categorical variables as absolute percentages. Mood´s median test, Kruskal-Wallis test and chi-squared (χ^2^) (or Fisher test if sample size was smaller than 20 or if any value was smaller than 5) were used, respectively, to compare continuous and categorical variables. Incidence of hyper- (PaCO_2_< 30 mmHg) and hypoventilation (PaCO_2_> 50 mmHg) in the different therapeutic groups was compared. Spearman´s Rho test was used to assess correlation between continuous variables. Statistical significance was defined as P<0.05.

## Results

We studied 46 piglets between 1 and 2 months of age weighing between 9 and 11 kg. They were randomized into three groups according to the breath rate during resuscitation: Group 1) 10 rpm (15 piglets); Group 2) 20 rpm (16 piglets); Group 3) 30 rpm (15 piglets). Main baseline characteristics of the 3 groups are described in S1 Table. There were no differences in the time to cardiac arrest: 7.0 (6.5–8) minutes in group 1; 7.1 (6–7.8) minutes in group 2 and 7.0 (6.5–8.4) minutes in group 3, (P = 0.85). Table 1 shows the main characteristics of the 3 groups before starting CPR. Forty-two piglets (91.3%) had non-shockable rhythms 10 minutes after cardiac arrest, with no significant differences between groups: 14 (93.3%), 15 (93.8%) and 13 (86.7%), respectively (P = 0.74).

**Table 1:** Comparison between main variables at baseline (10 minutes after remove piglets from ventilator).

Variable	10 rpm median (IQR)	20 rpm median (IQR)	30 rpm median (IQR)	P
SAP (mmHg)	20 (6–23)	19 (12–32)	22 (12.5–33)	0.34
DAP (mmHg)	13 (9.5–35.5)	10 (8.5–14)	11 (7.7–13.7)	0.21
MAP (mmHg)	12 (6–14.5)	12 (10–15.5)	16 (10.5–21.5)	0.50
CVP (mmHg)	12 (7–14)	10 (8–12)	9 (6–10)	0.25
t-SatO_2_ (%)	33 (15–48)	38 (23–51.5)	15.5 (15–47.2)	0.09
Cerebral rSO_2_ (%)	29.5 (18–34.7)	38 (23–51.5)	15.5 (15–47.2)	0.52
Splanchnic rSO_2_ (%)	0 (0–1.2)	0 (0–2)	0 (0–6.7)	0.81
Carotid blood flow(lpm)	1.2 (1–1.5)	1.6 (1.3–1.9)	1.2 (1–1.5)	0.07
Temperature (°C)	37.1 (36.4–37.4)	37.1 (36.2–38.4)	36.9 (36.3–37.7)	0.92
Arterial pH	7.14 (7.08–7.22)	7.09 (7.04–7.16)	7.13 (7.09–7.18)	0.44
PaCO_2_ (mmHg)	78 (70–90)	88 (74.7–97.5)	85 (75–97)	0.63
PaO_2_ (mmHg)	14 (11–16)	14.5 (7.2–17.7)	10 (8–17)	0.63
HCO_3_ (mEq/L)	20.3 (19.5–22.9)	20.1 (18–21.3)	22 (19.6–22.9)	0.25
S_a_O_2_ (%)	9 (5–12)	9 (3–11)	5 (4–11)	0.28
Lactic acid (mmol/L)	5.3 (5–7)	6.3 (5.3–7.2)	5.6 (4.4–6.1)	0.43
S_v_O_2_ (%)	7 (4–12)	7 (3–11)	8 (5–10)	0.94

rpm: respiration per minute; IQR: interquartile range; FC: cardiac frequency; bpm: beats per minute; SAP: systolic artery pressure; DAP: diastolic artery pressure; MAP: mean artery pressure; CVP: central venous pressure; t-SatO_2_: transcutaneous oxygen saturation; rSO2: regional oxygen saturation; lpm: litres per minute; Vt: tidal volume; FiO_2_: inspired oxygen fraction; PaCO_2_: arterial CO_2_ pressure; PaO_2_: arterial O_2_ pressure; HCO_3_: bicarbonate; S_a_O_2_: arterial O_2_ saturation; S_v_O_2_: venous O_2_ saturation.

ROSC was achieved in 22 piglets (47.8%) after CPR. The percentage of ROSC was higher in the 30 rpm group (60%), than in the 10 rpm (37.5%) and 20 rpm (46.6%) groups, but differences were not statistically significant (P = 0.51).

**Fig 1:**
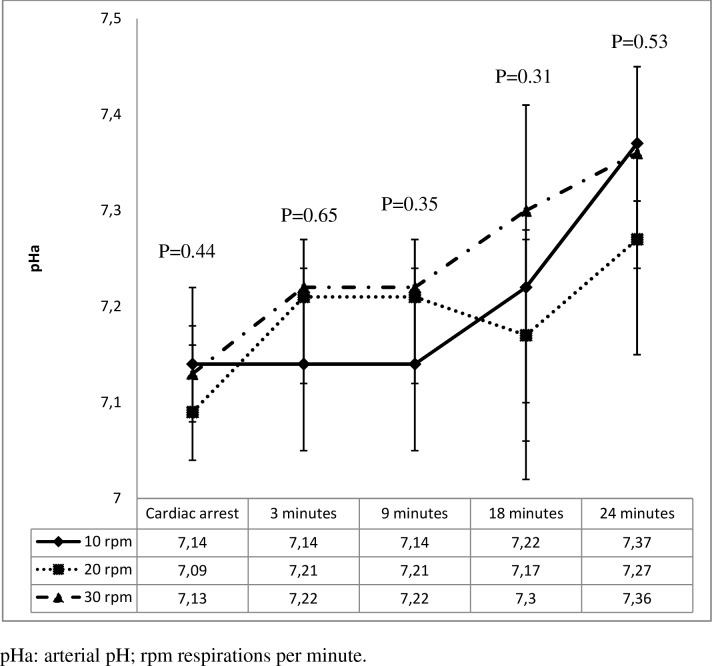
Arterial pH values during resuscitation.

**Fig 2:**
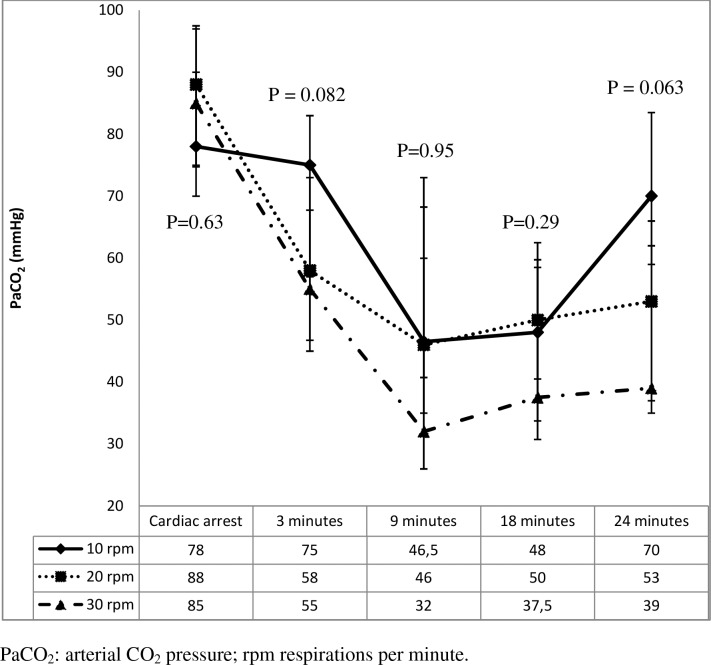
Arterial PCO_2_ values (mmHg) during resuscitation.

**Fig 3:**
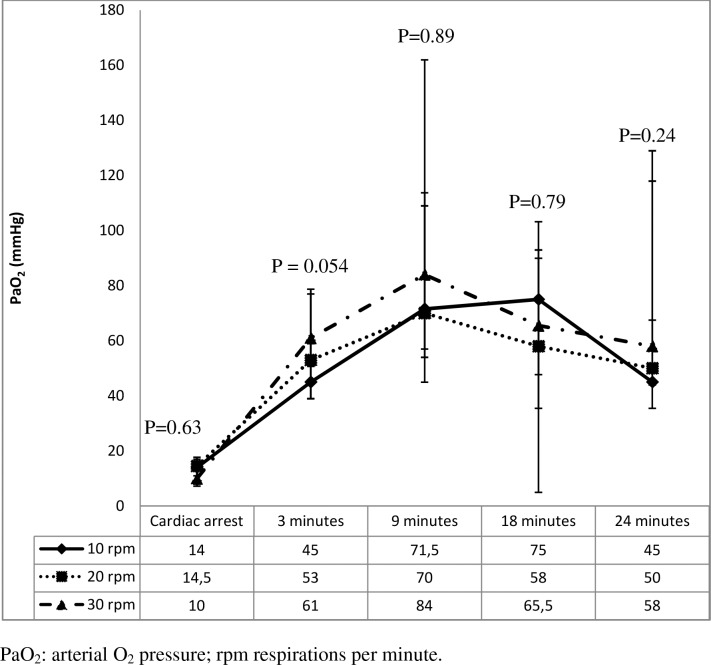
Arterial PO_2_ values (mmHg) during resuscitation.

**Fig 4:**
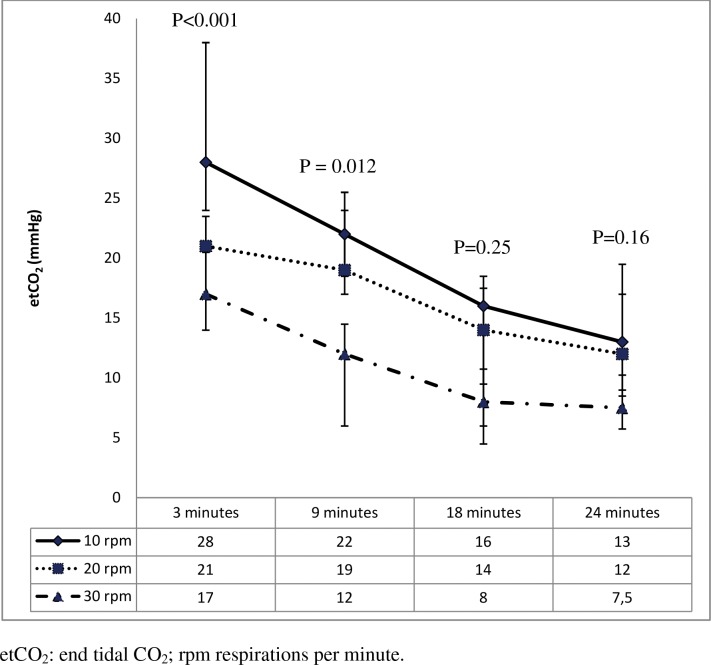
End-tidal CO_2_ values during resuscitation.

**Fig 5:**
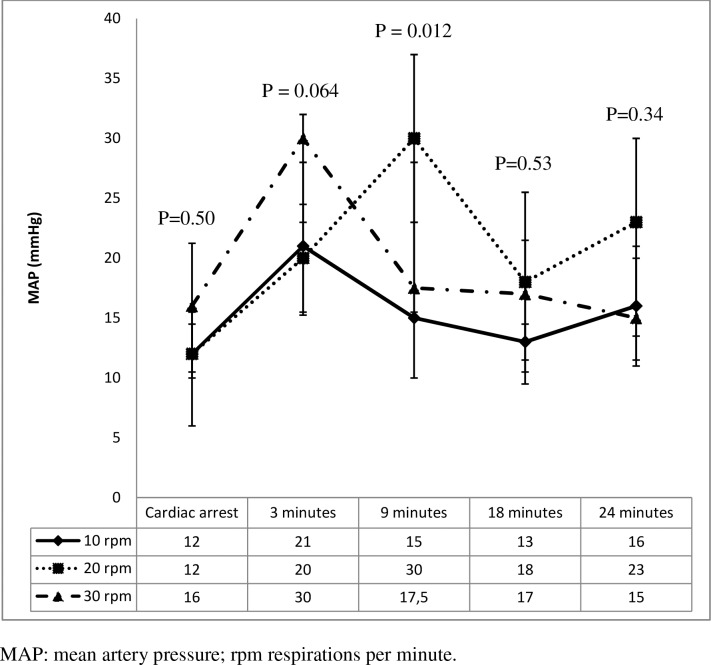
Mean artery pressure values during resuscitation.

Figs 1–5 shows the evolution of pH, PaO_2_ and PaCO_2_ throughout the experiment. Fig 1 shows how pH increased over the course of CPR in all the groups. PaCO_2_ decreased over the first 9 minutes of CPR in all groups. The greater difference is observed at 3 minutes of CPR: 55 mmHg (30 rpm group), 58 mmHg (20 rpm) and 75 mmHg (10 rpm), P = 0.08. Fig 2 shows how PaCO_2_ remained stable thereafter in groups 2 and 3 whereas it continued to rise in group 1 (10 rpm). Such a difference was most significant at 24 minutes after CPR (P = 0.06). Group 3 had the highest percentage of hyperventilated piglets (PaCO_2_<30 mmHg) at 9 minutes of CPR and the lowest percentage of hypoventilated patients at 24 minutes of CPR, as shown in Table 2.

**Table 2:** Hyper (PaCO_2_< 30 mmHg) and hypoventilated (PaCO_2_> 50 mmHg) piglets between the 3 groups along the hole resuscitation.

Time	Hyperventilated N/total				Hypoventilated N/total			
	10 rpm	20 rpm	30 rpm	P	10 rpm	20 rpm	30 rpm	P
3 minutes	0/15	0/15	0/14	1	12/15	10/15	8/14	0.41
9 minutes	0/10	1/11	3/7	0.03	3/10	4/11	3/7	0.86
18 minutes	0/9	1/10	0/6	0.45	4/9	5/10	1/6	0.39
24 minutes	0/9	0/9	0/5	1	8/9	5/9	1/5	0.03

PaCO2: arterial CO_2_ pressure; rpm: respirations per minute.

PaO_2_ increased significantly over the first 9 minutes of CPR and then dropped modestly in all groups. After 3 minutes of CPR, PaO_2_ was higher in group 3 than in groups 2 and 1 (61 mmHg, 53 mmHg and 45 mmHg, respectively); P = 0.05. There were no other significant differences between groups over the course of the study (Fig 3).

We compared MAP, carotid blood flow, cerebral SO_2_ and PaO_2_ between hyperventilated (PaCO_2_<30 mmHg), normoventilated (30–50 mmHg) and hypoventilated (> 50 mmHg) animals. PaO_2_ was significantly higher in hyperventilated (PaO_2_ 153 mmHg) than in normoventilated (79 mmHg) and hypoventilated (47 mmHg) piglets, but no significant differences were found in MAP, carotid blood flow or cerebral SO_2_ (Table 3).

**Table 3:** Variables comparison between ventilation status.

Variable	Hyperventilated (PaCO_2_< 30 mmHg)median (IQR)	Normoventilated (PaCO_2_ 30–50 mmHg)median (IQR)	Hypoventilated (PaCO_2_>50 mmHg)median (IQR)	P
MAP (mmHg)	19 (11.5–26.5)	20 (13–15.5)	20 (12–29)	0.91
Cerebral rSO_2_(%)	15 (14–15)	22 (15–34)	30 (15–55)	0.14
Carotid blood flow (lpm)	15.5 (10–15.5)	7 (1.2–13.2)	6 (1–13)	0.32
PaO_2_ (mmHg)	153 (103–190.5)	79 (58–96)	47 (40.2–60.7)	<0.001

PaCO2: arterial CO_2_ pressure; IQR: interquartile range; MAP: mean artery pressure; rSO2: regional oxygen saturation; lpm: litres per minute; PaO_2_: arterial O_2_ pressure.

There was a moderate correlation between PaO_2_ and pH (r = 0.514, P<0.001) as well as a moderate inverse correlation between PaO_2_ and PaCO_2_ (r = -0.694, P<0.001). A moderate correlation was also found between MAP and carotid blood flow (r = 0.468, P<0.001) (Table 4).

**Table 4:** Correlation between gasometrical values and MAP, carotid blood flow and cerebral rSO_2_.

Variables	PaO_2_	PaCO_2_	pH
	r P	r P	r P
PaO_2_ (mmHg)	*	- 0.694 (< 0.001)	0.514 (< 0.001)
PaCO_2_ (mmHg)	- 0.694 (< 0.001)	*	- 0.762 (< 0.001)
pH	0.514 (< 0.001)	- 0.762 (< 0.001)	*
MAP (mmHg)	0.039 (0.69)	- 0.06 (0.95)	- 0.225 (0.02)
Carotid blood flow(lpm)	0.022 (0.86)	- 0.081 (0.52)	- 0.029 (0.82)
Cerebral rSO_2_ (%)	- 0.134 (0.32)	0.287 (0.03)	- 0.227 (0.09)

MAP: mean artery pressure; rSO2: regional oxygen saturation; PaO_2_: arterial O_2_ pressure. PaCO2: arterial CO_2_ pressure; lpm: litres per minute.

Fig 4 shows that group number 3 (30 rpm) had significantly lower values of etCO_2_ than the other groups at 3 and at 9 minutes of CPR. Fig 5 shows a significant increase in MAP after 3 minutes of CPR followed by a progressive drop thereafter. MAP in the 10 rpm group was lower than in the other two groups at 3 minutes (P = 0.06) and 9 minutes (P = 0.01) of CPR. Diastolic arterial pressure (DAP) was higher in the 20 rpm group at 9 minutes of CPR than in the other groups. The rest of parameters did not show any statistically significant differences (S2 Table).

## Discussion

Our study is, to our knowledge, the first to analyze the effect of different RR during CPR on oxygenation, ventilation, haemodynamics, tissue perfusion and ROSC in a paediatric animal model of asphyxial cardiac arrest.

The results from this study offer some valuable information:

In the first place, haemodynamic, respiratory and tissue perfusion parameters improve during the first 9 minutes of CPR but then progressively deteriorate despite delivering good-quality CPR. ROSC was achieved mostly in the first 10 to 12 minutes of CPR. Only one piglet achieved ROSC after 12 minutes of CPR. This fact has also been observed in clinical studies [32,33] and other paediatric experimental animal models [7,30,31,34], which highlights the importance of accurately performing high quality resuscitation during the first minutes of CPR.

Secondly, piglets that were ventilated at a higher respiratory rate had lower PaCO_2_ over the course of CPR. EtCO_2_ was also lower in 30 rpm group (Fig 4) but etCO_2_ reflects not only ventilation but pulmonary blood flow. The percentage of hyperventilated piglets was higher in the 30 rpm group whereas the percentage of hypoventilated animals was higher in the 10 rpm group.However, hyperventilation risk with 30 rpm is lower than hypoventilation with 10 rpm. Some clinical studies in children show that both hyper- as well as hypoventilation during the first hour after ROSC are associated with higher mortality rates [25], but there are no clinical studies analyzing ventilation and mortality during CPR.

Animals in the 30 rpm group had a tendency to better oxygenation values during the first 10 minutes of CPR, which is the period of time in which ROSC is mainly achieved. This difference in oxygenation did not achieve statistical significance, but it may be relevant in clinical practice as the most frequent cause of cardiac arrest in children is asphyxia and thus hypoxia [2,5,8–15,34].

On the other hand, hyperventilated piglets with PaCO_2_<30 mmHg had significantly higher values of PaO_2_ with no differences in MAP, carotid blood flow or cerebral SO_2_ than the rest of piglets. Furthermore, there was a direct correlation between more ventilation (higher pH and lower PaCO_2_) and better oxygenation (PaO_2_). This is an important fact, as it suggests that, in asphyxial cardiac arrest, a higher RR results in more ventilation and better oxygenation as it improves gas exchange.

In the fourth place, performing CPR with higher RR than what international guidelines recommend, but which are more similar to normal and physiologic RR for paediatric patients, did not affect haemodynamic parameters at all during CPR. Moreover, piglets with higher RR had slightly higher MAP than those ventilated with 10 rpm. This fact supports previous results from this same animal model showing that CPR with ventilation as opposed to chest compressions only does not negatively affect haemodynamics during CPR [6,7]. Although MAP depends more on compressions quality than on RR, higher RR achieve better systemic and probably coronary ventilation and oxygenation improving haemodynamics at some point, but studies analyzing this specific effect are required.

Finally, animals receiving higher RR show a tendency (although not statistically significant) towards greater ROSC rates than the other groups. Nevertheless, studies with a bigger sample size are required in order to assess the influence of RR on ROSC.

Our study has several limitations. In the first place, even though our asphyxial paediatric cardiac arrest model has been validated and is very similar to what happens in human paediatric patients, results from experimental animal studies must be interpreted with caution. In the second place, sample size is probably insufficient to find statistically significant differences in ROSC, ventilation and oxygenation. Nevertheless, it offers some important information for further experimental animal models with a bigger sample size in order to analyze the influence of RR during CPR on ROSC.

In the third place, differences in oxygenation and MAP may be due not only to RR, but to the quality of manual chest compressions. Depth of chest compressions was not measured, but they were always performed by the same medical team, swapping the member providing chest compressions every 3 minutes to guarantee good-quality compressions. Frequency of chest compressions was tailored with a metronome.

Finally, our study analyzes the influence of RR during advanced CPR, where the patient is already intubated and chest compressions do not need to be interrupted to deliver breaths. Thus, our results cannot be fully extrapolated to non-intubated patients receiving basic life support.

## Conclusions

According to our model of asphyxial cardiac arrest, oxygenation, ventilation and global haemodynamics improve during the first 9 minutes of CPR, which is also when the highest percentage of ROSC is achieved. Nevertheless, all these parameters worsen beyond that point in time. Our study confirms the hypothesis that higher RR achieve better oxygenation and ventilation without affecting haemodynamics, carotid blood flow or cerebral oxygenation. Even though there was a positive tendency, we cannot conclude that a higher RR is associated with better ROSC rates.

Our results provide a basis for further experimental and clinical studies to assess the effect of higher-than-recommended RR during CPR in children.

## Supporting Information

S1 Table Comparison between main variables just before remove piglets from ventilator. (DOCX)

S2 Table Comparison between main variables during resuscitation. (DOCX)
