# Supplementary material for: Different Respiratory Rates during Resuscitation in a Pediatric Animal Model of Asphyxial Cardiac Arrest
Source: PLoS One. 2016 Sep 12;11(9):e0162185. doi: 10.1371/journal.pone.0162185 (PMC5019379; doi:10.1371/journal.pone.0162185)
Supplement: S1 Table — (DOCX) [file pone.0162185.s001.docx]

**S1 Table. Comparison between main variables just before remove piglets from ventilator.**

| Variable | 10 rpm  median (IQR) | 20 rpm  median (IQR) | 30 rpm  median (IQR) | P |
| --- | --- | --- | --- | --- |
| Weight (kg) | 10 (9-11) | 9.5 (9-10) | 9 (8-11) | 0.50 |
| Size (cm) | 68 (65-73) | 68 (66-72) | 69 (66-72) | 0.91 |
| CF  (bpm) | 103 (89-119) | 123.5 (100-144.5) | 102 (88-147) | 0.43 |
| SAP (mmHg) | 114 (105-120) | 112 (104.5-125.2) | 107 (98-114) | 0.38 |
| DAP (mmHg) | 53 (49.5-67.5) | 67 (56.5-73.2) | 56 (48-63) | 0.26 |
| MAP (mmHg) | 78 (71-89) | 83 (72-92.7) | 74 (69-86) | 0.44 |
| CVP (mmHg) | 4 (3-6) | 4 (2.2-6) | 5 (3-6) | 0.54 |
| t-SatO_2_ (%) | 100 (96-100) | 100 (98-100) | 100 (97-100) | 1 |
| Cerebral rSO_2_ (%) | 62 (45.5-65.5) | 62 (51.7-67.7) | 55 (43-68) | 0.28 |
| Splanchnic rSO_2_ (%) | 60 (53.5-66) | 59 (51.5-64.2) | 54 (50.5-57) | 0.06 |
| Carotid blood flow (lpm) | 46 (38-55) | 53 (41.2-63.2) | 55 (37-70) | 0.25 |
| Tisular blood flow (lpm) | 14.9 (9.7-18) | 12.2 (8.2-25) | 11.3 (7.5-14.7) | 0.55 |
| Temperature (ºC) | 37.5 (36.9-37.9) | 37.5 (36.7-38.3) | 37.3 (36.6-37.7) | 0.55 |
| Inspiratory Vt (mL) | 115 (89-120) | 110 (97.7-120) | 110 (95-120) | 0.77 |
| Expiratory Vt (mL) | 105 (87.2-116.2) | 100.5 (96.7-120) | 110 (85-110) | 0.86 |
| FiO_2_ (%) | 45 (40-55) | 45.5 (43.2-51.7) | 44 (40-52) | 0.81 |
| etCO_2_ (mmHg) | 35 (33-36) | 35 (33.2-39) | 36 (33-38) | 0.54 |
| Arterial pH | 7.46 (7.45-7.5) | 7.47 (7.42-7.5) | 7.48 (7.44-7.5) | 0.93 |
| PaCO_2_ (mmHg) | 42 (37-44) | 39 (38-44.7) | 41 (39-47) | 0.63 |
| PaO_2_ (mmHg) | 162 (129-191) | 163.5 (138.2-194.7) | 155 (103-187) | 0.63 |
| HCO_3_ (mEq/L) | 29.2 (28.8-30.3) | 29 (27.3-30.1) | 29.8 (28.4-30.2) | 0.20 |
| S_a_O_2_ ( %) | 100 (99-100) | 100 (99-100) | 99 (98-100) | 1 |
| Lactic acid (mmol/L) | 0.9 (0.8-1.2) | 0.9 (0.5-1.3) | 0.7 (0.5-0.9) | 0.27 |
| S_v_O_2_ (%) | 82 (70-86) | 81 (76.2-85) | 79 (70-79) | 0.03 |

rpm: respiration per minute; IQR: interquartile range; FC: cardiac frequency; bpm: beats per minute; SAP: systolic artery pressure; DAP: diastolic artery pressure; MAP: mean artery pressure; CVP: central venous pressure; t-SatO_2_: transcutaneous oxygen saturation; rSO2: regional oxygen saturation; lpm: litres per minute; Vt: tidal volume; FiO_2_: inspired oxygen fraction; etCO_2_: end tidal CO_2_; PaCO2: arterial CO_2_ pressure; PaO^2^: arterial O_2_ pressure; HCO3: bicarbonate; S_a_O_2_: arterial O_2_ saturation; S_v_O_2_: venous O_2_ saturation.
