# Supplementary material for: Different Respiratory Rates during Resuscitation in a Pediatric Animal Model of Asphyxial Cardiac Arrest
Source: PLoS One. 2016 Sep 12;11(9):e0162185. doi: 10.1371/journal.pone.0162185 (PMC5019379; doi:10.1371/journal.pone.0162185)
Supplement: S2 Table — (DOCX) [file pone.0162185.s002.docx]

**S2 Table. Comparison between main variables during resuscitation.**

| **Variables** | | **3 minutes**  median (IQR) | **9 minutes**  median (IQR) | **18 minutes**  median (IQR) | **24 minutes**  median (IQR) |
| --- | --- | --- | --- | --- | --- |
| SAP (mmHg) | | | | | |
|  | 10 rpm | 46 (38-67) | 36 (20-65) | 30 (23-46.5) | 40 (28-75) |
|  | 20 rpm | 39 (29.5-59) | 57 (42-71) | 52 (23.5-65) | 50 (35.5-63) |
|  | 30 rpm | 61 (44-78) | 48 (36.5-60.2) | 30 (25.5-56.5) | 36.5 (27-57.7) |
|  | P | 0.36 | 0.37 | 0.85 | 0.40 |
| DAP (mmHg) | | | | | |
|  | 10 rpm | 11.5 (6.2-29.5) | 6 (2.2-14) | 6.5 (5.2-9.2) | 7 (4-10) |
|  | 20 rpm | 19 (9-18) | 18 (14-25) | 7 (4-12.5) | 7 (3.5-20) |
|  | 30 rpm | 15 (8-19) | 8 (3-11.2) | 7 (5-12.5) | 7.5 (3.7-15.7) |
|  | P | 0.27 | 0.01 | 0.69 | 0.96 |
| cerebral rSO_2_ (%) | | | | | |
|  | 10 rpm | 23 (15.5-43) | 24 (15-24) | 22 (15-22) | 29 (24-52) |
|  | 20 rpm | 46.5 (18.7-63.2) | 28.5 (18-53.2) | 15 (15-15) | 15 (15-15) |
|  | 30 rpm | 24 (15-52.5) | 24 (14.7-83.7) | 15 (15-29.2) | 15 (15-29.2) |
|  | P | 0.32 | 0.87 | 0.51 | 0.21 |
| Splanchnic rSO_2_ (%) | | | | | |
|  | 10 rpm | 29 (25-44) | 33 (28-42) | 31 (27-47.5) | 34 (30-43) |
|  | 20 rpm | 30 (24-35.7) | 31 (19.7-34.7) | 36 (26-39) | 34 (21-38.7) |
|  | 30 rpm | 32 (28-37) | 32 (27-38) | 31 (23.25-38) | 32 (23.5-34.5) |
|  | P | 0.85 | 0.67 | 0.82 | 0.21 |
| Carotid blood flow (lpm) | | | | | |
|  | 10 rpm | 8 (3-14.5) | 8 (1.5-12.2) | 2 (1-2) | 6.5 (2-8.7) |
|  | 20 rpm | 5 (0-23) | 10 (0-18) | 4 (1-10) | 5.5 (1.2-12) |
|  | 30 rpm | 7 (5-16) | 6 (2-6) | 14.5 (4.2-18.7) | 1 (0-1) |
|  | P | 0.59 | 0.69 | 0.32 | 0.88 |
| Lactic acid (mmol/L) | | | | | |
|  | 10 rpm | 5.8 (5.1-6.8) | 6.8 (6.3-7.9) | 7.6 (7-9.3) | 8.5 (7.7-9.9) |
|  | 20 rpm | 6.8 (6.2-7.6) | 8.3 (7.2-9.1) | 9.5 (8.4-10.8) | 10.5 (8.5-11.5) |
|  | 30 rpm | 6.2 (5.6-6.5) | 7.7 (7.1-8) | 10.1 (8.2-10.7) | 11.1 (8.9-11.6) |
|  | P | 0.39 | 0.29 | 0.15 | 0.53 |
| S_a_O_2_ (%) | | | | | |
|  | 10 rpm | 67 (46-80) | 94 (70.2-96.7) | 93 (78-97) | 72 (41-88.7) |
|  | 20 rpm | 79 (56-92) | 85 (71-97) | 83 (68-96.7) | 89 (65-97) |
|  | 30 rpm | 87.5 (75.7-92.5) | 95 (57-99) | 90 (64.2-97) | 93 (76-99) |
|  | P | 0.05 | 0.50 | 0.79 | 0.66 |
| S_v_O_2_ (%) | | | | | |
|  | 10 rpm | 14.5 (8.5-37) | 16.5 (7.5-38) | 20 (13.5-39.5) | 17 (12.5-31.5) |
|  | 20 rpm | 17 (7-34) | 18 (6-29) | 12.5 (7.5-22.7) | 14 (8.5-54.5) |
|  | 30 rpm | 15 (10.7-40) | 20 (11-23) | 12.5 (7.7-17.2) | 15 (10.5-24) |
|  | P | 0.84 | 0.72 | 0.15 | 0.82 |

IQR: interquartile range; rpm: respiration per minute; SAP: systolic artery pressure; DAP: diastolic artery pressure; MAP: mean artery pressure; rSO2: regional oxygen saturation; lpm: litres per minute; S_a_O_2_: arterial O_2_ saturation; S_v_O_2_: venous O_2_ saturation.
